# Comparison of levonorgestrel level and creamatocrit in milk following immediate versus delayed postpartum placement of the levonorgestrel IUD

**DOI:** 10.1186/s12905-021-01179-7

**Published:** 2021-01-21

**Authors:** Niaree G. Hopelian, Rebecca G. Simmons, Jessica N. Sanders, Katherine Ward, Sabrina Malone Jenkins, Eve Espey, David K. Turok

**Affiliations:** 1 The University of Utah School of Medicine, 30 N 1900 E, 2B200, Salt Lake City, UT 84132 USA; 2 Division of Family Planning, Department of Obstetrics and Gynecology, The University of Utah School of Medicine, 30 N 1900 E, 2B200, Salt Lake City, UT 84132 USA; 3 The University of Utah College of Nursing, 10 S 2000 E, Salt Lake City, UT 84112 USA; 4 Division of Neonatology, Department of Pediatrics, The University of Utah School of Medicine, 295 Chipeta Way, Salt Lake City, UT 84108 USA; 5 Department of Obstetrics and Gynecology, The University of New Mexico School of Medicine, Albuquerque, NM 87131 USA; 6 Present Address: Department of Psychiatry, Neuropsychiatric Institute, University of Illinois, Chicago, 912 S Wood St, Chicago, IL 60612 USA

**Keywords:** Intrauterine device, Levonorgestrel, Postpartum contraception, Human milk, Breastfeeding, Creamatocrit

## Abstract

**Background:**

Breastfeeding and postpartum contraception critically influence infant and maternal health outcomes. In this pilot study, we explore the effects of timing and duration of postpartum levonorgestrel exposure on milk lipid and levonorgestrel content to establish baseline data for future research.

**Methods:**

This sub-study recruited a balanced convenience sample from 259 participants enrolled in a parent randomized controlled trial comparing immediate to delayed (4–8 weeks) postpartum levonorgestrel IUD placement. All planned to breastfeed, self-selected for sub-study participation, and provided the first sample at 4–8 weeks postpartum (before IUD placement for the delayed group) and the second four weeks later. We used the Wilcoxon rank sum (inter-group) and signed rank (intra-group) tests to compare milk lipid content (creamatocrit) and levonorgestrel levels between groups and time points.

**Results:**

We recruited 15 participants from the immediate group and 17 from the delayed group with 10 and 12, respectively, providing both early and late samples. Initially, median levonorgestrel concentration of the immediate group (n = 10) (32.5 pg/mL, *IQR*: 24.8, 59.4) exceeded that of the delayed group (n = 12) (17.5 pg/mL, *IQR*: 0.0, 25.8) (*p* = 0.01). Four weeks later, the values aligned: 26.2 pg/mL (*IQR*: 20.3, 37.3) vs. 28.0 pg/mL (*IQR*: 25.2, 40.8). Creamatocrits were similar between both groups and timepoints.

**Conclusions:**

Immediate postpartum levonorgestrel IUD placement results in steady, low levels of levonorgestrel in milk without apparent effects on lipid content. These findings provide initial support for the safety of immediate postpartum levonorgestrel IUD initiation, though the study was not powered to detect noninferiority between groups.

*Trial Registration*: This randomized controlled trial was registered with ClinicalTrials.gov (Registry No. NCT01990703) on November 21, 2013.

## Background

Both breastfeeding and the use of postpartum contraception critically influence not only infant but also maternal health outcomes [1, 2]. Immediate postpartum initiation of highly effective, long-acting contraception eliminates the risk of early pregnancy and avoids the need to return to the clinic for (typically less comfortable) intrauterine device (IUD) placement [3]. Understanding the effect of immediate postpartum hormonal contraception on breastfeeding through objective assessments of milk production and exogenous progestogen content can inform counseling and decision-making. However, we lack information assessing hormonal levels and milk content in people who are breastfeeding while using hormonal contraceptives. In particular, prior research has not assessed the cumulative effect of timing or duration of levonorgestrel exposure via IUD on levels in human milk or the consequence of duration on milk fat levels.

Four decades ago, researchers published results using accurate but logistically challenging, radioimmunoassay measurements to assess levonorgestrel levels in milk from women with high-dose levonorgestrel IUDs [4]. While radioassays are highly accurate, radiolabels confer a short shelf life and limit the use of this test [5]. Measuring hormone levels in milk is time consuming due to the slow extraction procedure, therefore there is a paucity of information on hormones in breastmilk. The recent development of a commercially-available, enzyme-linked immunoassay (EIA) for quantitative measurement of levonorgestrel in water, saliva, milk, urine and extracted serum, plasma and feces provides new opportunities for research laboratories to assess levonorgestrel exposure. The EIA kit detects very small amounts of levonorgestrel in multiple media and from multiple species [6]. Our pilot research represents the first comparative clinical trial assessing variable timing of levonorgestrel exposure in human milk using this EIA kit.

Hormonal contraceptive users who are breastfeeding also seek information about changes in milk content that may be related to exogenous hormonal exposure. Creamatocrit is a simple measure of lipid content in milk that can easily conducted with basic laboratory equipment [7]. Although the creamatocrit lacks detail on all energy components of human milk, it focuses on fat content, which supplies the majority of calories and thus energy. It has been particularly valuable as a crude assessment of energy content for studying milk for low-birth-weight infants in developing countries. Creamatocrit is closely correlated with the gold standard for measuring lipid content in human milk, the gravimetric method (r^2^ = 0.99) [8].

Assessment of change in milk composition, including creamatocrit levels, can help determine whether timing of hormonal exposure plays a factor in outcomes when comparing early initiation of progestogen-containing postpartum long-acting reversible contraception to a delayed postpartum insertion. While human milk creamatocrit was prospectively evaluated in 1,322 exclusively breastfed infants [9], contraceptive exposure was not typically reported. A small study of immediate versus delayed contraceptive implant use showed no difference in lactogenesis or creamatocrit at 6 weeks postpartum [10]. However, there is still a gap in comparisons of change in creamatocrit over time after immediate postpartum IUD insertion.

The purpose of this study therefore is to assess the role of timing of levonorgestrel IUD placement in postpartum outcomes. We compare the levonorgestrel and creamatocrit concentrations between women randomly assigned to immediate (less than 30 min) postpartum levonorgestrel IUD insertion to those with delayed (4–8 week) insertion. We hypothesized that women receiving an immediate postpartum levonorgestrel IUD would have an early presence of levonorgestrel compared to those receiving their IUD later and that follow up levonorgestrel levels would be similar between groups when both had had the IUD for at least four weeks. Also, we anticipated that milk fat content would not differ by levonorgestrel exposure.

Using an enzyme-linked immunoassay for levonorgestrel and traditional creamatocrit measurements, we sought exploratory data to assess differences in milk levonorgestrel and lipid content over time to provide point estimates for future research on exogenous progestogen and creamatocrit lipid levels among women receiving an early versus delayed postpartum levonorgestrel IUD.

## Methods

### Design

This nested, prospective, longitudinal sub-study is an exploratory secondary analysis derived from the Breastfeeding Levonorgestrel IUD Study (BLIS) randomized controlled noninferiority trial. BLIS compared breastfeeding continuation at eight weeks postpartum between women who received immediate (within 30 min) post-placental levonorgestrel IUD placement and delayed (4–8 weeks postpartum) placement. Thus, criteria for eligibility for this sub-study were prior consent and enrollment in the parent study. Parent study details, including randomization allocation and other relevant recruitment data, have been previously reported [11]. All sub-study participants enrolled in Salt Lake City, Utah. The Institutional Review Board at the University of Utah approved the study [IRB#_00062844]. This sub-study adheres to the CONSORT guidelines for randomized trials.

**Fig. 1:**
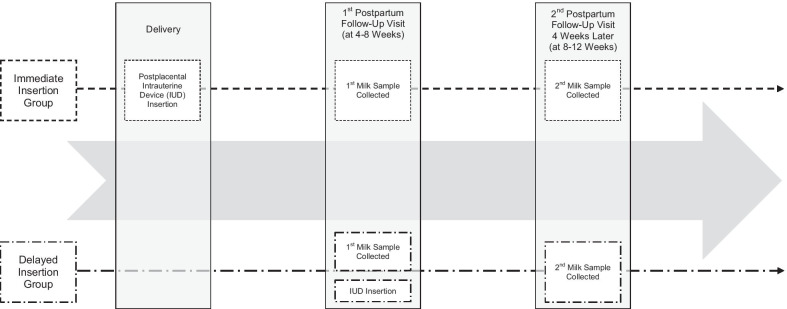
Timeline of participant intrauterine device (IUD) placement and milk collection by assignment group

From January 2014 through November 2016, research personnel used medical records to approach potential participants at multiple clinical sites in Salt Lake City. All participants had plans to breastfeed and a stated interest in the levonorgestrel IUD. Study participants provided written consent and opted into the sub-study using a checkbox on the parent study's consent documents. Figure 1 details the study timeline by assignment group. We asked all sub-study participants to provide self-expressed milk samples at both the first postpartum visit (at 4–8 weeks) and the second four weeks later. In the delayed group, the first milk sample was obtained prior to IUD placement. In this exploratory study, we compared both levels of levonorgestrel and creamatocrit in the milk samples of women who received IUDs at different timepoints.

### Sample

For this sub-study, a balanced convenience sample self-selected to participate from both groups of the 259 women who enrolled in the parent study. The parent study offered enrollment to people experiencing uncomplicated pregnancies, fluent in English or Spanish, aged 18–40 years old, who desired the levonorgestrel IUD as their postpartum method of contraception, planned to breastfeed, and agreed to the randomization of levonorgestrel IUD placement timing. The parent study's pre-specified exclusion criteria included delivery before 37 weeks gestational age, chorioamnionitis, postpartum hemorrhage, contraindications to levonorgestrel IUD placement, and medical complications of pregnancy that could affect breastfeeding. The main study consent form included an explanation of the sub-study and a checkbox for people to decline or agree to participate in this sub-study. Sub-study participants in both the immediate and delayed placement groups returned for a follow-up visit four weeks after IUD placement. We planned to cease recruitment when we obtained two samples (initial and four-week follow-up) from 12 participants in each group. Sample size was not determined by statistical power but rather to create point estimates and confidence intervals for creamatocrit and levonorgestrel levels, which can inform future work. We oversampled by 25% in case of loss to follow-up [12].

### Creamatocrit measurement

Participants provided two mid-feeding milk samples (10 ml each) at a 4–8 week postpartum clinic visit and second samples at home approximately four weeks later. For those in the delayed group, initial sample collection occurred at the same clinic visit as IUD placement. All other samples were collected after IUD placement, as in Fig. 1. Research personnel transported samples to an − 80 °C freezer within one hour of expression. Later, a research team member (NGH) masked to group assignment thawed each sample and measured lipid levels using the creamatocrit method twice and reported mean values for analysis [7]. Samples were returned to the − 80 °C freezer to await the subsequent levonorgestrel assay.

### Levonorgestrel assay

We shipped frozen milk samples to Arbor Assays in Ann Arbor, Michigan, for analysis. There, technicians processed each sample using Arbor Assay DetectX Levonorgestrel Enzyme Immunoassay Kit instructions [6]. Samples were run in duplicate with a known levonorgestrel standard. SoftMax® 4 parameter logistic fitting software (Molecular Devices, San Jose, CA) was used to calculate results. See Supplement A for assay details and commentary.

### Data analysis

We blinded statisticians to group assignments for data analysis. Using Stata 15 statistical software (StataCorp LP, College Station, TX USA), we conducted the Wilcoxon rank sum (inter-group) and signed rank (intra-group) tests to compare levonorgestrel levels and creamatocrit between groups and times. Non-parametric tests were selected due to the small sample size and lack of normal data distribution. We also conducted a sensitivity analysis to compare outcomes among those who experienced IUD expulsions (and subsequent replacement) (expulsion in delayed group = 1 [5%], expulsion in immediate group = 2 [9%]) and no expulsion (n = 19, [86%]).

## Results

### Participant characteristics

Table 1 presents participant characteristics. In total, 32 participants provided an initial milk sample at the first postpartum visit. Of these, 22 (immediate = 12, delayed = 10) provided a second sample, and we limit analyses to this group. Ten participants (immediate = 5, delayed = 5) did not provide a second sample for reasons including discontinuing breastfeeding and IUD expulsion without replacement.

**Table 1:** Demographics of BLIS participants providing milk samples, by randomization arm (*N* = 22)

Variables	Early insertion	Delayed insertion
	*n* = 12	*n* = 10
	*n* (%)	*n* (%)
Age^a^		
18–24	4 (33)	2 (22)
25–29	4 (33)	3 (33)
30–34	3 (25)	2 (22)
35–39	1 (8)	2 (22)
Full-time employment		
Yes	1 (8)	1 (10)
No	11 (92)	9 (90)
History of obesity		
Yes	2 (20)	0
No	8 (80)	10 (100)
Gravidity		
None	0	0
1	1 (8)	0
≥ 2	11 (92)	10 (100)
History of prior breastfeeding		
Yes	11 (92)	9 (90)
No	1 (8)	1 (10)
Initial breastfeeding duration goal		
< 6 months	1 (8)	1 (10)
> 6 months	11 (92)	9 (90)

BLIS stands for **B**reastfeeding **L**evonorgesterel **I**ntrauterine Device **S**tudy

^a^One participant in the delayed insertion group did not provide age

### Levonorgestrel concentrations

At the first postpartum visit, the median levonorgestrel concentration of the immediate group was 32.5 pg/mL (*IQR*: 24.8, 59.4), and the delayed group was 17.5 pg/mL (*IQR*: 0.0, 25.8), (*p* = 0.01). Four weeks later, the values aligned: 26.2 pg/mL (*IQR*: 20.3, 37.3) in the immediate group and 28.0 pg/mL (*IQR*: 25.2, 40.8) in the delayed group. No difference was found in the immediate group's levonorgestrel concentrations between postpartum visits (*p* = 0.38). The sensitivity analysis described above did not significantly alter results.

### Creamatocrit

We observed similar median creamatocrit values at the initial (4–8 weeks) postpartum visit [immediate = 5.9% (*IQR*: 2.8%, 8.1%); delayed = 5.1% (*IQR*: 3.4%, 7.4%)] and four weeks later [immediate = 4.7% (*IQR*: 3.9%, 7.7%); delayed = 3.9% (*IQR*: 3.6%, 4.9%)]. Additionally, intragroup median creamatocrits were similar between the two time points for the immediate or delayed groups. Sensitivity analysis did not significantly alter results.

## Discussion

This exploratory study provides point estimates for milk levonorgestrel levels and lipid levels in postpartum individuals initiating levonorgestrel IUD contraception immediately and 4–8 weeks later. We found that women receiving delayed IUD placement eventually have similar levonorgestrel milk levels and similar milk lipid content compared to those receiving immediate postpartum IUD placement. Our findings align with previous research, suggesting the temporal stability of levonorgestrel levels in milk [4].

It is unclear why most participants in the delayed group had trace amounts of levonorgestrel in their initial milk sample before the placement of a levonorgestrel IUD. However, presence of exogenous hormones in drinking water suggests there may be baseline environmental contamination [13], which could cause this. Nevertheless, a study examining levonorgestrel levels in human milk from those taking levonorgestrel-containing oral contraceptive pills found that the level in levonorgestrel in milk was between 250 and 500 pg/mL, over ten times the amount found in our study [14]. Notably, our results are consistent with those from the assay development, which also identified trace amounts of levonorgestrel in urine samples of individuals (including males) not using a levonorgestrel-containing product [6]. While these findings may represent contamination or cross-reactivity of the assay, they may also represent classification bias. Further research investigating background levonorgestrel levels in human milk may help clarify the significance of this finding.

Study limitations include small sample size and limited power to detect small differences between groups, between primiparous and multiparous women, across BMI, across body fat distribution, and across time by the number of weeks postpartum. Furthermore, even though participants had enrolled in a randomized controlled trial, their election to participate in this sub-study introduces the potential for selection bias. While ethnicity likely does not affect lipid or hormone levels in human milk, all participants identified as Latina, and this could potentially limit external validity. Also, we lack information on neonatal and infant outcomes, including growth and development data. Initially, we planned to obtain infant plasma levonorgestrel levels; however, no participants agreed to an infant blood draw.

Moreover, we did not measure total milk volumes, which theoretically, can be influenced by hormonal contraception. At follow-up, many participants reported changes in breastfeeding behavior such as bottle feeding, supplementation, or exclusive artificial milk feeding. Breastfeeding behavior affects milk composition, and these changes may have influenced lipid levels. Lastly, creamatocrit has traditionally been useful as a proxy measure of milk energy content. However, we now know that various factors, including the time of day collected, time since the last feeding, whether fresh or stored, and whether foremilk or hindmilk may influence creamatocrit [15]. Thus, a single sample limits our ability to assess this variability.

## Conclusion

Overall, our study provides evidence that immediate postpartum levonorgestrel IUD placement results in steady, low levels of levonorgestrel in milk without apparent effects on lipid content. These findings add to the growing body of literature supporting the safety of immediate postpartum levonorgestrel IUD initiation. While not powered to detect noninferiority, our study presents baseline data to inform future research. Future studies examining differences between immediate and delayed contraceptive use should assess time-based meaningful outcome changes to capture clinically significant differences.

## Data Availability

The datasets used and analyzed during the current study are available from the corresponding author on reasonable request.

## Abbreviation

IUD: Intrauterine device
